# New Perspective on Anorexia Nervosa: Tryptophan-Kynurenine Pathway Hypothesis

**DOI:** 10.3390/nu15041030

**Published:** 2023-02-18

**Authors:** Charl Alberts, Maja Owe-Larsson, Ewa M. Urbanska

**Affiliations:** 1 Department of Experimental and Clinical Pharmacology, Medical University of Lublin, Jaczewskiego 8B, 20-059 Lublin, Poland; 2 Department of Histology and Embryology, Center of Biostructure Research, Medical University of Warsaw, Chałubińskiego 5, 02-004 Warsaw, Poland; 3 Laboratory of Center for Preclinical Research, Department of Experimental and Clinical Physiology, Medical University of Warsaw, Banacha 1B, 02-097 Warsaw, Poland; 4 Laboratory of Cellular and Molecular Pharmacology, Department of Experimental and Clinical Pharmacology, Medical University of Lublin, Jaczewskiego 8B, 20-059 Lublin, Poland

**Keywords:** anorexia nervosa, L-kynurenine, kynurenic acid, quinolinic acid, aryl hydrocarbon receptor, kynurenine aminotransferase, exercise

## Abstract

Anorexia nervosa (AN), affecting up to 4% of all females and 0.3% of all males globally, remains the neuropsychiatric disorder with the highest mortality rate. However, the response to the current therapeutic options is rarely satisfactory. Considering the devastating prognosis of survival among patients with AN, further research aimed at developing novel, more effective therapies for AN is essential. Brain and serum tryptophan is mostly converted along the kynurenine pathway into multiple neuroactive derivatives, whereas only 1–2% is used for the synthesis of serotonin. This narrative review provides an update on the experimental and clinical research data concerning the metabolism of tryptophan along the kynurenine pathway in anorexia nervosa based on the available literature. We propose that in AN, lower levels of L-kynurenine and kynurenic acid result in diminished stimulation of the aryl hydrocarbon receptor, which could contribute to abnormally low body weight. The impact of L-kynurenine supplementation on anorexia in animal models and the effects of changes in tryptophan and downstream kynurenines on the clinical progression of AN require further investigation. Moreover, prospective clinical studies on larger cohorts of restrictive and binge-eating/purging AN patients and assessing the potential benefit of L-kynurenine as an add-on therapeutic agent, should follow.

## 1. Introduction

Anorexia nervosa (AN), commonly manifesting with self-starvation, extreme anxiety, hyperactivity, and amenorrhea, remains the psychiatric disorder with the highest mortality rate. It affects up to 4% of all females and 0.3% of all males globally, and recent studies indicate an increasing incidence in younger people aged <15 years [1,2]. A recent review analyzing the data from 2013 to 2022 demonstrates that 5.5–17.9% of young women and 0.6–2.4% of young men have experienced a DSM-5 eating disorder by early adulthood. Lifetime DSM-5 anorexia nervosa was reported by 0.8–6.3% of women and 0.1–0.3% of men, with gender and sexual minorities at particularly high risk. Emerging studies from Eastern Europe, Asia, and Latin America show a similarly high prevalence. During the COVID-19 pandemic, the incidence of eating disorders increased [2]. A large meta-analysis of 42 anorexia-related mortality studies (with follow-up periods from 1.7 to 33 years and a total of 3006 subjects) revealed a crude death rate in AN of 5.9%, with an aggregate mortality rate of 5.6% per decade. Suicide rates were 200-fold more likely among patients with AN than in the general population [3]. The majority of AN outpatients are readmitted five or more years after presentation [4]. Some 20-year follow-up studies revealed that full recovery was achieved in 27.5–50.6% of patients and that 15.6–17.5% had died from causes related to the disease [5,6]. 

AN is a disorder with a very complex etiology, including congenital and environmental factors. Patients typically present with major depressive disorder or generalized anxiety disorder, among others, as psychiatric comorbidities [7]. Behavioral dysfunction includes pathological reward and eating habits, impaired appetite, altered impulse control, neuroticism, anxiety, and lowered mood. Low body mass is achieved with pathological dieting, purging, binge eating, or excessive physical exercise. Accordingly, AN is categorized into two major subtypes: restrictive and binge-eating/purging [8,9]. In the brain, profound changes in the connectivity and neurotransmission within the dopaminergic and serotoninergic systems are frequently observed [7]. Morphological assessments indicate brain atrophy involving gray and white matter volume reductions [10,11]. However, the findings are inconsistent, as the location and extent of changes are highly variable. Following the body weight restoration, brain volume reductions may be reversible, and it is unclear whether long-term morphological complications persist in severe AN [12,13].

The disturbed function of the serotoninergic system in the etiology of AN is substantiated by a number of experimental and clinical data. However, the results are conflicting, and it is uncertain whether the changes are of a causative or symptomatic nature [14]. Serotonin is synthesized in the brain and periphery from tryptophan (TRP), yet only a minor part of the TRP pool enters this metabolic pathway. TRP depletion stimulated by stress and inflammation lowers peripheral and central serotonin levels [15], and a majority of research in AN focuses on this aspect of TRP metabolism. It was suggested that there is a correlation between pathological behavioral traits, such as anxiety, obsessions, or harm avoidance, and serotonin. Thus, starvation may be used by patients with AN to decrease dysphoria. On the other hand, the deficient serotoninergic transmission may result in a depressive mood and further aggravate the course of the disease [16].

Noteworthy, a majority of serum TRP is converted along the kynurenine pathway (KP) into multiple neuroactive derivatives termed collectively as kynurenines. Accumulated evidence clearly indicates the importance of bilateral interactions between TRP metabolism along the KP and inflammatory pathways [17]. Furthermore, the majority of brain kynurenines are generated within glial cells, and there is increasing evidence of the role of glial cells in the pathophysiology of eating disorders, including anorexia nervosa [18]. Yet, the potential contribution of disturbed TRP metabolism along the KP to the development of AN is poorly understood. Thus, we aimed to review the available data on TRP and its metabolism along the KP in AN. We searched articles published in English that appeared in Medline/Pubmed using key terms relating to anorexia nervosa, the metabolites of TRP along the KP, and the pathway enzymes. The accumulated evidence is scarce, yet it supports the notion that AN is an illness in which the deficiency of dietary TRP lessens not only the synthesis of serotonin but also hampers kynurenine synthesis. This, in turn, may further contribute to troubled emotions and disturbed eating behaviors. We highlight the possible association between glia, inflammation, and kynurenines in AN. The KP emerges as an important link integrating environmental and inflammation-related changes with the clinical course of AN.

## 2. The Kynurenine Pathway 

**Figure 1:**
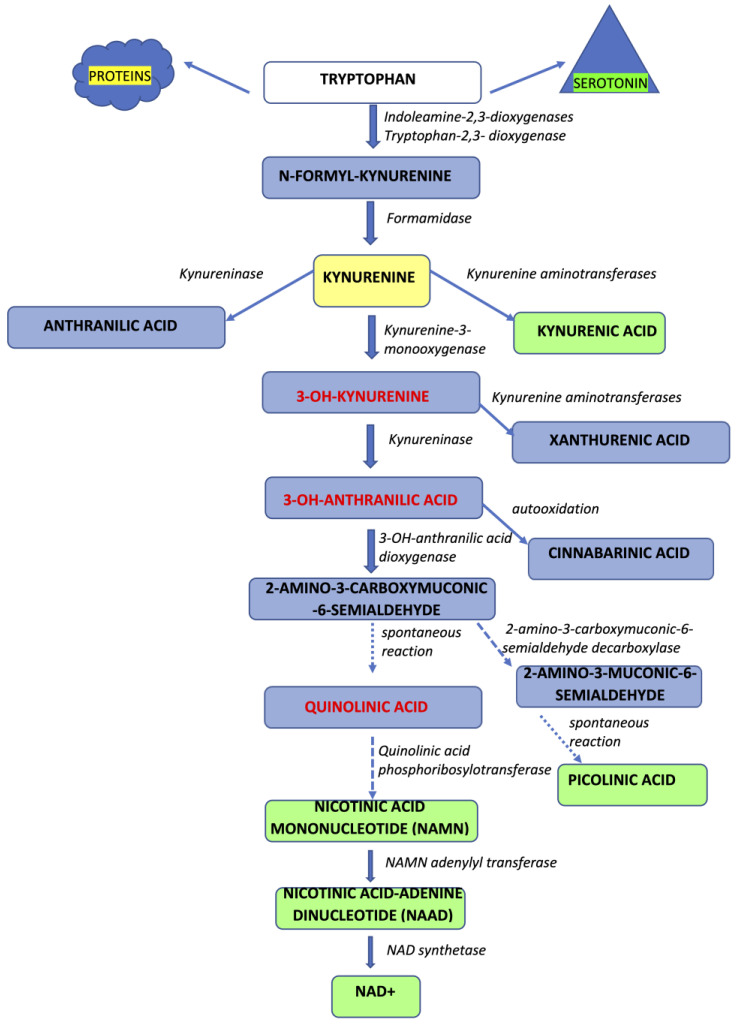
The scheme of tryptophan metabolism along the kynurenine pathway (KP). The majority of available serum tryptophan enters the KP. IDOs or TDO convert tryptophan into L-kynurenine, which is further catabolized by kynurenine aminotransferases to kynurenic acid, kynurenine-3-monooxygenase to 3-OH-L-kynurenine, or kynureninase to anthranilic acid. Kynurenine aminotransferases may convert 3-OH-L-kynurenine to xanthurenic acid or kynureninase to form 3-OH-anthranilic acid. Further, 3-OH-anthranilic acid is converted into quinolinic or picolinic acid by a series of enzymatic conversions.

In humans, TRP supplied with food is incorporated into structural and enzymatic proteins, serves as a source of serotonin and melatonin, or is converted along the kynurenine pathway (KP) into a number of bioactive molecules [19,20,21] (Figure 1).

Approximately 95% of serum TRP enters the KP, whereas only a minor proportion of this amino acid yields serotonin and other products. The first step of the pathway is catabolized by tryptophan 2,3-dioxygenase (TDO) or indoleamine 2,3-dioxygenases (IDO1 and IDO2), forming N-formylkynurenine, which is subsequently converted into L-kynurenine [19,20,21]. TDO, expressed mostly in hepatocytes, is strongly upregulated by tryptophan itself and glucocorticoids [22]. IDO-1 expression was detected in microglia, neurons, and astrocytes, as well as in macrophages, epithelial cells, and fibroblasts. During inflammation, either overt or low-grade, released cytokines such as interleukin-1 (IL-1), IL-6, interferon-γ (IFN-γ), or tumor necrosis factor-α (TNF-α) and they act as molecular signals activating IDO via pathways involving a signal transducer and an activator of transcription (STAT)-1, IFN-regulatory factor-1, p38 mitogen-activated protein kinase (MAPK), and NF-*κ*B [23,24,25]. However, due to rather low activities of IDOs and TDO in the brain, a substantial amount of the central pool of L-kynurenine originates from peripheral sources (60–70%), whereas the remaining 30–40% is produced in situ [26]. An increase in L-kynurenine local biosynthesis has been observed in neuroinflammation and is presumed to occur partly due to the immune stimulation of IDO [17,25]. Inflammation has been linked to altered eating habits under pathological conditions, with cytokines such as, e.g., IFN-γ modulate food intake regulation and wasting [27,28]. 

L-kynurenine is further catabolized into various metabolites displaying numerous and often opposing biological properties. Kynurenines were shown to exert pro- and anti-inflammatory effects, to either cause cytotoxicity or prevent it, or to act as free radical scavengers or a source of free radicals. Considering its central position in the KP, L-kynurenine serves as a switch point, directing the metabolic fate of TRP in three directions. 

The first route of L-kynurenine metabolism, considered neuroprotective, is catalyzed by four distinct aminotransferases (KATs I-IV), generating kynurenic acid (KYNA). KAT I [glutamine transaminase K (GTK)/cysteine conjugate beta-lyase (CCBL) 1], KAT II [aminoadipate aminotransferase (AADAT)], KAT III (CCBL2), and KAT IV [glutamic-oxaloacetic transaminase (GOT) 2/mitochondrial aspartate aminotransferase (mASPAT)] were detected in rodent and human brains [29]. Due to its polar structure, KYNA does not easily cross the blood–brain barrier. In contrast, TRP and L-kynurenine are actively transported from the periphery into the brain via the large neutral amino acid (LNAA) transport system [26]. Furthermore, diverse substrate profiles, dissimilar optimal pH ranges, and various region-specific activities are characteristic for KATs. Overall, the local regulation of KYNA synthesis seems finely tuned and precisely controlled [21]. 

The second route yields 3-OH-kynurenine through the activity of kynurenine 3-monooxygenase (KMO). Indirectly, the activity of KMO, which displays a much lower K_m_ value for L-kynurenine, also impacts the synthesis of KYNA. 3-OH-kynurenine is further metabolized to quinolinic acid (QUIN) through initial conversion by kynureninase into 3-hydroxyanthranilic acid (3-HAA) and subsequent breakdown into unstable ACMS by 3-hydroxyanthranilic acid oxygenase [20,21]. QUIN is a source of nicotinamide adenine dinucleotide^+^ (NAD^+^), which is essential in cellular metabolism. The third possible route of L-kynurenine metabolism results from kynureninase-mediated conversion, which yields anthranilic acid, which may further generate 3-hyroxyanthranilic acid. Enzymatic conversion by KATs can occur for 3-OH-kynurenine into xanthurenic acid [30]. 

Experimental data from in vitro and in vivo studies clearly demonstrated the neuroprotective properties of KYNA and the neurotoxic effects of such derivatives as 3-OH-kynurenine or QUIN. Furthermore, a number of generated kynurenines also manifest potent immunomodulatory properties [17]. L-kynurenine, KYNA, and xanthurenic acid act as ligands of the aryl hydrocarbon receptor (AhR), known to play an essential role in limiting immune response and the induction of immune tolerance, partly through the conversion of naive T cells to the anti-inflammatory Treg phenotype [17,31,32].

KYNA is considered a unique TRP metabolite due to its pleiotropic effects [21,33]. This compound was initially identified as an endogenous nonselective antagonist of all 3 ionotropic excitatory amino acid receptors: N-methyl-D-aspartate (NMDA), α-amino-3-hydroxy-5-methyl-4-isoxazole propionic acid ((R,S)-AMPA), and kainate type. KYNA displays the highest affinity for the strychnine-insensitive glycine site of the NMDA receptor [21]. Furthermore, KYNA, apart from being an agonist of AhR, is also an agonist of the orphan receptor GP35 and an antagonist of α-7 nicotinic cholinergic receptors, although data concerning the latter effect are unclear [21,33,34]. Moreover, KYNA plays a role as a direct reactive oxygen species (ROS) scavenger [21,33].

Another product of the KP, QUIN, is a potent agonist of excitatory amino acid receptors of the NMDA type. It is produced in the brain mostly by microglia and macrophages [35]. QUIN is well known to cause neuronal cell death through excitotoxic processes. Furthermore, it may increase free radical production by inducing NOS activity in astrocytes and neurons, inhibit succinate dehydrogenase, thus impairing the mitochondrial respiratory chain, or induce cytoskeletal disruption [35]. Thus, increased availability of L-kynurenine may result in two opposite scenarios: neuroprotective and neurotoxic. Cellular protection would be linked mostly to the higher availability of KYNA. KYNA would be produced at the expense of the other two routes, and an increase in the KYNA/3-OH-kynurenine and KYNA/QUIN ratios would follow.

The hypothesis linking neuroinflammation with increased metabolism of TRP along the KP and its shift towards neurotoxic kynurenines is well substantiated [17,36,37]. An increasing number of data suggest that an elevated expression of pro-inflammatory cytokines, such as TNFα, IFNγ, IL-1, and IL-6, may favor catabolic reactions and cachexia. IL-1, IFNγ, and TNFα were shown to affect the hypothalamic neurons implicated in the regulation of eating behavior and appetite [38]. It is unclear whether the disturbed cytokine profile detected in patients with AN results from impaired nutrition, psychopathological, and neuroendocrine factors or inherited genetic disturbances [38]. In most of the prospective studies, initially altered cytokine serum levels returned to normal values after re-nutrition [38], thus suggesting their temporary nature. However, the sequel of changes linking altered immune responses with pathological eating behaviors is still unclear. We suggest that in AN, the environment promoting neuronal damage and oxidative stress, resulting in sustained neuroinflammation, may cause mood disturbances and dysfunction of hypothalamic anorexigenic pathways, in part through the alterations in kynurenine synthesis and function.

## 3. Tryptophan and Kynurenines in Anorexia Nervosa

In the following sections, we present the available data on TRP and its metabolites along the KP in AN (Table 1).

### 3.1. Tryptophan

The role of TRP in the control of food intake and its correlation with brain serotonin was the subject of several studies in experimental animals [56]. In animals receiving protein-poor but rich in carbohydrate foods or injected with insulin, plasma and brain TRP levels increased with a subsequent transient rise in serotonin levels [57]. However, when even larger elevations of plasma TRP are generated by the ingestion of protein-containing diets, no changes in the brain levels of TRP and serotonin occur [58]. The plasma amino acid pattern modulates the uptake of TRP into the brain through competition with other LNAA for their transporter. Paradoxically, an increase in brain TRP is evoked by dietary carbohydrates, whereas protein-rich foods containing TRP fail to do so. Most probably, insulin markedly lowers the plasma level of LNAA, which allows more TRP to enter the brain. Dietary proteins raise plasma TRP levels, but due to the rather low content of TRP, this rise is small in comparison with that of more abundant amino acids, such as leucine, isoleucine, and valine, which compete for the LNAA transporter [59]. 

Following an acute IL-1 injection in rats, food intake significantly declined, which was paralleled by a decrease in plasma-free TRP and an increase in plasma LNAA. In contrast, IL-1 significantly increased the concentration of TRP in rodents’ CSF [60]. Similarly, after peripheral or intra-hypothalamic administration of cytokines, feeding behavior is hindered via a mechanism linked with an enhanced expression and release of leptin [61,62]. 

The above changes are characteristic only of short-lasting changes in eating patterns and seem to serve as a protective mechanism, allowing the individual to maintain relatively undisturbed serotoninergic neurotransmission. During extended starvation, an actual decline in the brain’s TRP and serotonin levels is frequently observed. A study comparing rats subjected to either a 4-day restricted diet or a 4-day restricted diet and subsequent feeding for 2 days revealed that starvation lowers brain TRP levels [39,40]. The same group reported that prolonged starvation results in disparate effects concerning TRP levels in male and female rats. Restricted feeding decreased plasma TRP concentration in starved and starved/re-fed females and starved males. Hypothalamic TRP levels were lower in starved and starved/re-fed female rats and starved/re-fed males. Serotonin decreased in both starved and starved/re-fed male and female rats. Food restriction decreased serotonin levels in other brain areas of male but not female rats [41]. 

Clinical evidence mostly demonstrates the deficiency of TRP and LNAA in the blood of AN patients. Therefore, it is generally assumed that during either evident AN or restored nutrition, the TRP level merely represents nutritional status in anorectics. However, the results of the clinical studies are puzzling because both normal and reduced levels of TRP were detected. Considering that TRP and LNAA compete for the transporter that shifts amino acids across the blood–brain barrier, the peripheral TRP/LNAA ratio can indicate the quantities of TRP that enter the brain [63]. Thus, it is often measured as a peripheral parameter reflecting the status of brain TRP availability. However, the results are also perplexing, as normal, lower, or higher TRP/LNAA ratios were found in AN.

In a group of 35 patients with AN, plasma TRP levels did not differ from controls [43]. Similarly, the study in 16 anorectic patients revealed that blood TRP and LNAA levels were within normal ranges. There was no correlation between the TRP/LNAA ratios and the Hopkins Symptom Checklist (HSCL) and Eating Disorders Inventory (EDI). However, the TRP/LNAA ratio seemed to be higher in patients exercising excessively, with a more severe catabolic status. The ratio was inversely correlated with the body mass index, body fat, muscle mass, daily energy intake, and daily TRP intake. Thus, it was suggested that the TRP/LNAA ratio is not controlled only by the diet but is associated with the status of catabolism [44]. Likewise, a cross-sectional study including 59 dialyzed patients, of which 13 manifested poor appetite and malnutrition, revealed that the plasma levels of free TRP, LNAA, and TRP/LNAA ratios were similar between anorectic and well-nourished patients [45]. Similarly, a study of 20 female patients with restrictive AN revealed no changes in serum TRP compared to 24 control individuals [53]. 

In contrast, others showed a decrease in TRP levels in the blood of anorectic patients. A study of 26 female AN patients and 15 control subjects revealed that plasma TRP is significantly lower in low-weight patients [46]. Data from 19 anorectic patients, 10 of whom presented with associated bulimic features, demonstrated lower TRP or TRP/LNAA ratios as opposed to 12 healthy controls [46]. Similarly, a study comprising 32 patients who recovered from severe AN, 32 acutely underweight patients with AN, and 32 healthy individuals showed significantly lower plasma levels of TRP among anorectics [47]. Serum levels of TRP in 16 patients with AN were significantly lower than in control groups [50]. A study of 34 patients with acute AN, 19 weight-recovered patients, and 35 healthy control women revealed lower plasma TRP and detected a positive correlation between TRP and S100B, a small calcium-binding protein [64].

During the recovery period, plasma TRP usually increases in AN. A study of 14 women with symptomatic AN, 14 women who recovered from AN, and 15 healthy control individuals revealed that both groups of anorectic women had significantly higher mean baseline TRP/LNAA ratios compared with the control [51]. In a cohort of 26 female patients with AN and 15 control subjects, plasma TRP, LNAA, and the TRP/LNAA ratio increased significantly during refeeding [46]. Similarly, in 42 anorectic patients, the levels of serotonin, TRP, LNAA, and the TRP/LNAA ratio were considerably lower than in the control, both at admission and after refeeding. During the refeeding, the levels of measured metabolites increased significantly in comparison with admission but did not reach control values [48]. 

No difference in the CSF TRP level was found between 33 anorexic and 14 healthy women [65]. Similarly, a study analyzing the levels of kynurenines in the CSF samples obtained from 10 medication-free female patients with AN, 22 normal-weight bulimia nervosa patients, and 8 healthy females revealed that the level of TRP was not altered in the AN group [52]. The TRP level was negatively associated with the body mass index (BMI) in AN [52].

### 3.2. L-Kynurenine and Kynurenic Acid

Experimental data support the notion that kynurenines may modify feeding behavior. A number of studies were performed on animal models of anorexia. One of the models employs the administration of polyinosino:polycytidylic acid (Poly I:C) viral mimic that is a synthetic double-stranded RNA. It is a strong inducer of an immune response that produces profound anorexia in mice [66,67]. Poly I:C-treated mice maintain muscle strength and motor coordination, along with the ability to climb and rear; however, they manifest weight loss, which seems to result from a decreased drive to eat [66,68]. It was demonstrated that Poly I:C evokes a potent rise in L-kynurenine levels and an increase in the L-kynurenine/TRP ratio [66]. Thus, activation of the KP seems to be linked with anorectic behavior. Metabolomic analysis of 391 metabolites during hunger and satiety in *Drosophila melanogaster* revealed that, in response to eating, metabolic profiles change in a distinct way. L-kynurenine depletion was detected in the heads of flies on a high-sugar diet, supporting the view that the L-kynurenine level is correlated with the brain energy state and feeding behavior [69]. These observations suggest that higher L-kynurenine levels may contribute to decreased food intake, whereas lower L-kynurenine is associated with feeding. However, it cannot be excluded that the observed changes are not the cause but the result of a disturbed eating pattern.

In fact, others reported that in *Caenorhabditis elegans* displaying food-related behavioral plasticity, fasting depletes L-kynurenine and KYNA levels but not TRP levels [42]. In *C. elegans*, NKAT-1 and NKAT-3 are homologous to mammalian KATs. In *nkat-1* mutants, depletion of KYNA during fasting was shown to be a regulatory factor needed for the hyperactivation of feeding in *C. elegans* when they re-encounter food. KYNA depletion leads to reduced stimulation of NMDA receptors on neurons that communicate with serotonergic sensory neurons. Upon refeeding, KYNA levels are eventually replenished, ending the elevated feeding period. These data suggest the role of KYNA as a measure conveying the peripheral metabolic state that controls serotonin signaling in *C. elegans* [42]. A similar mechanism may also exist in humans and participate in the regulation of food intake during anorectic conditions.

So far, only three clinical studies have evaluated the downstream metabolites of TRP along the KP in AN. A study of 10 medication-free female patients with AN revealed reduced CSF levels of KYNA but not of kynurenine among underweight anorectic patients as opposed to normal-weight females. KYNA deficiency normalized as the body weight increased [52]. A quarter of a century later, the peripheral levels of kynurenines were investigated in 20 female patients with restrictive AN (mostly drug-free, all during the first episode of the disease) and compared with 24 controls [53]. We detected neither changes in serum L-kynurenine, KYNA, nor in the ratios TRP/L-kynurenine or L-kynurenine/KYNA. Thus, our investigation did not confirm the presence of KP changes in peripheral blood among AN patients, in contrast, to study in CSF [52]. However, their studies were performed in CSF samples, reflecting the condition of the TRP metabolism along the KP in the brain tissue. Very recently, DNA methylation profiles at promoter-associated CpG sites of the *SCL6A4* gene, encoding for the serotonin transporter (SERT), and the serum KYN/TRP ratio were studied in a cohort of eating disorders patients, including 45 patients with restrictive AN, 21 with purging AN, 21 with bulimia (BN), 31 with binge eating disorders, 23 with unspecified feeding or eating disorders, 14 with other specified eating disorders, and 34 healthy controls. Anorectic and bulimic patients did not manifest changes in L-kynurenine levels compared to controls. There was a small but significant increase in the TRP level among purging AN, as well as a small decrease in the L-kynurenine/TRP ratio [54]. Excessive physical activity in both groups of patients with AN resulted in a lower L-kynurenine level and higher TRP compared to non-exercising patients [54].

### 3.3. 3-Hydroxykynurenine and Quinolinic Acid

Neither 3-OH-kynurenine levels nor L-kynurenine/3-OH-kynurenine and KYNA/3-OH-kynurenine ratios were altered in the serum of 20 females with restrictive AN [53]. In 10 medication-free female patients with AN, CSF QUIN levels were within the control range, yet the ratio QUIN/KYNA was significantly higher during the underweight phase of AN [52]. Furthermore, a correlation between urinary cortisol excretion and the QUIN/KYNA ratio in CSF was observed. Considering that hypercortisolism can induce TDO activity and increase systemic production of kynurenines [70], this mechanism could be responsible for enhanced conversion of TRP towards QUIN. On the other hand, the effects of glucocorticoids on QUIN levels are not straightforward, as both a decrease or no change in QUIN levels were observed after glucocorticoid administration [71,72]. In contrast, it is well documented that immune stimulation and stress may increase the conversion of TRP along the branch of the KP leading to QUIN, with distinct effects in various structures of the brain [73,74]. Possibly, QUIN levels are strongly increased due to genetic errors prior to the overt development of AN. Then, during the fully active phase of AN, the attenuating effects of hypercortisolism could lower the QUIN level. However, considering the concomitant decrease of KYNA, the ratio QUIN/KYNA would still be higher, as observed [52].

### 3.4. Enzymes of the Kynurenine Pathway 

The results of Demitrack and co-workers indicated impaired KYNA generation in the CSF of AN patients with a concurrent lack of changes in the concentration of its direct bioprecursor, L-kynurenine [52]. Thus, it was suggested that KYNA deficiency occurred due to altered activity of biosynthetic enzymes, KATs [52]. The probable decline in KATs activity could result from deficient nutrition, including an inappropriate supply of enzymatic cofactors, such as vitamin B6 (pyridoxal 5’-phosphate) and pyruvate [52]. However, the authors did not measure the actual activity of KAT enzymes.

The expression of mRNA for *KAT1–3* was studied in 20 female patients with restrictive AN and in 24 controls [53]. The expression of the gene encoding *KAT3*, but not of the genes encoding *KAT1* and *KAT2,* was higher in 20 patients with restrictive AN. KYNA is produced mainly within astrocytes, primarily by KAT2 [75]. Interestingly, in animal models of AN, the density of astrocytic cells, as well as the expression of glial fibrillary acidic protein (GFAP), glutamate transporters (GLT-1 and GLAST), and glutamine synthetase, are reduced [18,76]. It was recently suggested that glial function is compromised by anorexia [77]. Therefore, it seems probable that dysfunctional glial cells produce lower quantities of KYNA. Lower KYNA levels in CSF would reflect region-specific and restricted to changes in TRP metabolism in the brain among AN patients. The peripheral metabolism of TRP may be affected differently and influenced by various factors able to restore/maintain KYNA levels within normal values.

Noteworthy, in C57BL/6 mice during activity-based anorexia, an additional physical exercise during the refeeding period resulted in an increased expression of muscle *KAT3* and *KAT4* mRNA levels, in contrast to re-fed mice without access to a running wheel [55]. Moreover, KYNA synthesis from L-kynurenine in muscle tissue was stimulated during the recovery from a malnourished state, but only in physically active animals [55]. Physical exercise was shown to stimulate the expression of muscle *KAT1, 3,* and *4* in humans [78]. Similarly, a significantly higher expression of the *KAT3* gene was found in a cohort of patients with restrictive AN who exercised excessively prior to their admission [53]. Thus, it is very likely that the observed increase in *KAT3* expression is a consequence of patients’ intense physical activity, which in turn may reverse the initial deficit of KYNA synthesis.

## 4. Dietary Interventions

The administration of TRP was evaluated as a means to affect the processes regulating food intake. A study in animals, either stress-free or subjected to short-duration immobilization stress, showed that TRP (300 mg/kg, orally) increased peripheral levels of serotonin and leptin while decreasing ghrelin levels in rats not exposed to stress [79]. There were no changes in 24-h cumulative food intake and elevated plus maze performance. In contrast, animals subjected to 2-h of stress manifested a decreased 24-h food intake, increased anxiety, lower serum serotonin, and increased leptin. TRP pre-treatment prevented the occurrence of behavioral and biochemical alterations [79]. Others have shown that subcutaneous application of TRP (100 mg/kg, for 2 days) increased both free and total TRP but did not change food intake, body weight, carcass adiposity, or leptin levels. Plasma-free TRP, but not total TRP, was significantly negatively correlated with food intake [80].

Intraperitoneal TRP administration after food deprivation evokes a hypothalamic serotonin increase, similar to exposure to the smell of food, eating a meal, or application of d-fenfluramine, as shown by a microdialysis study in rats. These data imply that TRP, among other factors, can be used to preferentially modify hypothalamic serotonin [81]. 

The beneficial effects of TRP were also shown in a rat model of immobilization stress-induced anorexia. TRP administration attenuated the impairment of food intake and ameliorated depressive symptoms [40]. Interestingly, TRP-fed animals did not manifest increased brain TRP, possibly due to a prominent peripheral metabolism. Alternatively, it can be assumed that as stress increases TRP hydroxylase activity, even a minor rise in brain TRP leads to its immediate conversion into serotonin and 5-hydroxyindoleacetic acid. Chronic intraperitoneal TRP administration attenuated the hyperactivity associated with a 38-day starvation in rats [82]. TRP treatment in starving rats normalized the declining levels of dopamine, serotonin, and their metabolites in the striatum [82]. 

The impact of TRP on eating habits in healthy and anorectic patients has been the subject of some studies. A high dose of TRP given to healthy individuals decreased the total caloric, carbohydrate, and protein intakes, whereas a smaller dose of TRP increased caloric and protein intakes but decreased carbohydrate intake [83]. Similarly, TRP administration to healthy volunteers in a high-protein serving was shown to decrease the intake of carbohydrates during the following free-choice meal [84]. A clinical randomized controlled trial in 29 healthy subjects allocated to a high- (*n* = 14) or low-protein diet (*n* = 15) for 2 weeks has shown a decreased plasma KYNA, but not TRP or L-kynurenine, on a low-protein diet. Plasma QUIN levels were below the detection limit. In urine, the levels of KYNA and QUIN, but not of L-kynurenine, were reduced on a low-protein diet. Surprisingly, metabolite changes were markedly different between human subjects and mice. The authors also conducted research on 20 wild-type FVB mice who were randomly assigned a high-protein or control diet for 21 days. In contrast to humans, a high-protein diet resulted in lower TRP and L-kynurenine levels than in controls. However, the authors did not assess the effect of a low-protein diet in experimental animals. Thus, it is rather difficult to draw a clear conclusion [85]. 

Another study investigated 22 healthy subjects (11 males and 11 females, aged 25.9 ± 4.2 years), and a total of 92 biomarkers were measured before a standardized meal as well as 30 and 120 min afterward. The only studied marker relevant to the KP was kynureninase. No changes were detected in its protein level, but a correlation with BMI was found [86]. The co-administration of a nutritional supplement containing 2,3 g of TRP with fluoxetine (5–26 weeks) was studied in a double-blind, placebo-controlled manner in a group of 26 subjects with AN. No effect of supplementation on weight gain, anxiety, or obsessive-compulsive symptoms in AN was observed [87].

As shown in a cohort of 42 individuals with AN, an increase in the TRP/LNAA ratio during refeeding correlated with a decrease in depressive symptoms [48]. On the other hand, increased anxiety was frequently linked with higher TRP levels. Intravenous TRP blunted growth hormone secretion and augmented the anxiety levels in AN [88]. Consequently, several studies have aimed at assessing the potential anxiolytic effects of TRP depletion. However, the results concerning the status of behavioral and biochemical parameters after TRP depletion in various neuropsychiatric conditions, such as depression, bulimia nervosa, autism, aggression, and substance dependence, are inconsistent [89]. 

In terms of AN, the data on TRP depletion are rather limited. The study performed on 14 women with symptomatic AN and 14 women who recovered from AN demonstrated that TRP depletion leads to a significantly greater reduction in the TRP/LNAA among anorectics. Patients from both AN groups also experienced a significant reduction in anxiety on the TRP depletion day compared with the placebo [51]. Acute TRP depletion in AN increased resting-state functional connectivity in patients recovered from AN (*n*= 22) in a similar manner to control individuals (*n* = 22) [90]. The same group reported that, despite the reduction of TRP/LNAA ratio, there was no difference in anxiety and mood between recovered AN (*n* = 22) and healthy controls (*n* = 25) exposed to TRP depletion [91]. Finally, based on functional magnetic resonance imaging, acute TRP depletion was shown to normalize the reward-related neural responses in recovered AN (*n* = 22) compared to controls (*n* = 25) [92].

Thus, the possibility of using TRP as a supplementary therapy in AN remains open and requires further detailed studies.

## 5. Concluding Remarks and Future Directions

Experimental and clinical research data indicate that TRP metabolism along the KP is disturbed in anorexia. However, the available research is limited, and the results are not clear-cut. Furthermore, the data originate mostly from studies in the peripheral compartment, and the analyses in the brain or CSF of patients with AN are scarce. 

The available data concerning the effects of TRP loading or depletion seem to support the hypotheses that, among individuals predisposed toward or recovered from AN and manifesting excessive serotoninergic transmission, the restricted TRP supply, through a reduction in serotonin availability, may alleviate dysphoric moods. 

Furthermore, careful interpretation of the evidence suggests that the central deficit of L-kynurenine supply as well as an impaired astrocytic production of KYNA are associated with AN. L-kynurenine and KYNA are both agonists of AHR. In experimental animals fed a Western diet, the AHR may disrupt fat metabolism and contribute to obesity [93,94]. Mice deficient in IDO1, an enzyme that metabolizes TRP, were resistant to obesity. At physiological levels, L-kynurenine was shown to activate AHR-directed luciferase expression. As enhanced IDO1 activity may increase L-kynurenine levels, this might activate the AHR and cause weight gain. In contrast, a deficiency of L-kynurenine and insufficient stimulation of the AHR may lead to weight loss [93,94]. Indeed, AHR-knockout mice tend to exhibit extreme physiologic abnormalities, including a reduction in body weight [32]. We propose that among AN patients manifesting lower levels of L-kynurenine and KYNA, the stimulation of AHR is impaired, which could contribute to the abnormally low body weight. 

In conclusion, there is a great need for basic science research assessing enzymes and metabolites of the KP, including their temporal pattern, in the ontogeny of AN. The impact of changed TRP and L-kynurenine levels on anorexia and the effects of alterations in other downstream kynurenines on the clinical progression of AN require further investigation. Furthermore, prospective clinical studies on larger cohorts of restrictive and binge-eating/purging AN patients, assessing the potential benefit of modulation of TRP and L-kynurenine levels as add-on therapy, should follow. Finally, detailed studies on the links between excessive physical activity and higher expression of KAT3 are needed. Considering the devastating prognosis for survival among patients with AN, further research aimed at developing novel, more effective therapies for AN is essential.

## Figures and Tables

**Table 1:** Tryptophan, kynurenines, and the kynurenine pathway enzymes in experimental and clinical studies on anorexia nervosa.

Metabolites/Enzymes	Conditions	Type of Samples	Results	Reference Number
Tryptophan	Four-day starvation in rats	Brain	↓	[39,40]
	Four-week starvation in rats	Plasma	↓	[41]
		Brain (hypothalamus)	↓	[41]
	*Caenorhabditis elegans*, in culture, 2 h fasting	Extracts from *C. elegans*	←→	[42]
	AN (*n* = 35)	Plasma	←→	[43]
	AN (*n* = 16)	Blood	←→	[44]
	Anorectic, dialyzed (*n* = 13)	Plasma	←→	[45]
	AN (*n* = 26)	Plasma	↓	[46]
	Acute AN (*n* = 32), Recovered AN (*n* = 32)	Plasma	↓	[47]
	AN (*n* = 42)	Plasma	↓	[48]
	AN (*n* = 19)	Whole blood	↓	[49]
	AN (*n* = 16)	Serum	↓	[50]
	Acute AN (*n* = 14), Recovered AN (*n* = 14)	Plasma	↓ acute AN vs. recovered ←→ vs. CTR	[51]
	Medication-free AN (*n* = 10)	CSF	←→	[52]
	Restrictive AN (*n* = 20)	Serum	←→	[53]
	Purging AN (*n* = 21)	Serum	↑	[54]
Tryptophan/LNAA ratio	AN (*n* = 16)	Blood	↑ in more severe catabolic status	[44]
	Anorectic, dialyzed (*n* = 13)	Plasma	←→	[45]
	AN (*n* = 19)	Whole blood	↓	[49]
	Acute AN (*n* = 14), Recovered AN (*n* = 14)	Plasma	↑	[51]
	AN (*n* = 42)	Plasma	↓	[48]
L-kynurenine	*Caenorhabditis elegans*, in culture, 2 h fasting	Extracts from *C. elegans*	↓	[42]
	Medication-free AN (*n* = 10)	CSF	←→	[52]
	Restrictive AN (*n* = 20)	Serum	←→	[53]
	Restrictive AN (*n* = 45), purging AN (*n* = 21)	Serum	←→	[54]
3-OH-kynurenine	*Caenorhabditis elegans*, in culture, 2 h fasting	Extracts from *C. elegans*	↓	[42]
	Restrictive AN (*n* = 20)	Serum	←→	[53]
Kynurenic acid	Medication-free AN (*n* = 10)	CSF	↓	[52]
	Restrictive AN (*n* = 20)	Serum	←→	[53]
Quinolinic acid	Medication-free AN (*n* = 10)	CSF	←→	[52]
*KAT1* gene expression	Restrictive AN (*n* = 20)	Serum	←→	[53]
*KAT2* gene expression	Restrictive AN (*n* = 20)	Serum	←→	[53]
*KAT3* gene expression	C57BL/6 mice, activity-based anorexia	Muscles	↑	[55]
	Restrictive AN (*n* = 20)	Serum	↑	[53]
*KAT4* gene expression	C57BL/6 mice, activity-based anorexia	Muscles	↑	[55]

↓—decrease; ↑—increase; and ←→—no change. AN—anorexia nervosa; CSF—cerebrospinal fluid; CTR—control; KAT—kynurenine aminotransferase; and LNAA—large neutral amino acids.

## Data Availability

No new data were created.
